# Genetic analysis of probable sleep bruxism and its associations with clinical and behavioral traits

**DOI:** 10.1093/sleep/zsad107

**Published:** 2023-04-26

**Authors:** Tommi Strausz, Satu Strausz, FinnGen, Tuula Palotie, Jari Ahlberg, Hanna M Ollila

**Affiliations:** Institute for Molecular Medicine Finland, Helsinki Institute of Life Science, University of Helsinki, Helsinki, Finland; Institute for Molecular Medicine Finland, Helsinki Institute of Life Science, University of Helsinki, Helsinki, Finland; Department of Genetics, Stanford University School of Medicine, Stanford, CA, USA; Department of Oral and Maxillofacial Diseases, Head and Neck Center, Helsinki University Hospital, Helsinki, Finland; Orthodontics, Department of Oral and Maxillofacial Diseases, Clinicum, Faculty of Medicine, University of Helsinki, Helsinki, Finland; Department of Oral and Maxillofacial Diseases, Head and Neck Center, Helsinki University Hospital, Helsinki, Finland; Institute for Molecular Medicine Finland, Helsinki Institute of Life Science, University of Helsinki, Helsinki, Finland; Broad Institute of MIT and Harvard, Cambridge, MA, USA; Center for Genomic Medicine, Massachusetts General Hospital, Boston, MA, USA; Anesthesia, Critical Care, and Pain Medicine, Massachusetts General Hospital and Harvard Medical School, Boston, MA, USA

**Keywords:** Sleep Bruxism, Genome-Wide Association Study, Population Register, FinnGen, Temporomandibular Joint Dysfunction Syndrome, Pain, Gastroesophageal Reflux, Obstructive Sleep Apnea

## Abstract

**Study Objectives:**

Sleep bruxism (SB) can cause damage on teeth, headache and severe pain affecting both sleep and daily functioning. Yet despite the growing interest into bruxism, the underlying clinically relevant biological mechanisms remain unresolved. The aim of our study was to understand biological mechanisms and clinical correlates of SB including previously reported disease associations.

**Methods:**

We used data from the FinnGen release R9 (*N* = 377 277 individuals) that are linked with Finnish hospital and primary care registries. We identified 12 297 (3.26%) individuals with International Classification of Diseases (ICD)-10 codes used for SB. In addition, we used logistic regression to examine the association between probable SB and its clinically diagnosed risk factors and comorbidities using ICD-10 codes. Furthermore, we examined medication purchases using prescription registry. Finally, we performed the first genome-wide association analysis for probable SB and computed genetic correlations using questionnaire, lifestyle, and clinical traits.

**Results:**

The genome-wide association analysis revealed one significant association: rs10193179 intronic to *Myosin IIIB* (*MYO3B)* gene. In addition, we observed phenotypic associations and high genetic correlations with pain diagnoses, sleep apnea, reflux disease, upper respiratory diseases, psychiatric traits, and also their related medications such as antidepressants and sleep medication (*p* < 1e-4 for each trait).

**Conclusions:**

Our study provides a large-scale genetic framework to understand risk factors for SB and suggests potential biological mechanisms. Furthermore, our work strengthens the important earlier work that highlights SB as a trait that is associated with multiple axes of health. As part of this study, we provide genome-wide summary statistics that we hope will be useful for the scientific community studying SB.

## Graphical Abstract

**Graphical Abstract:**
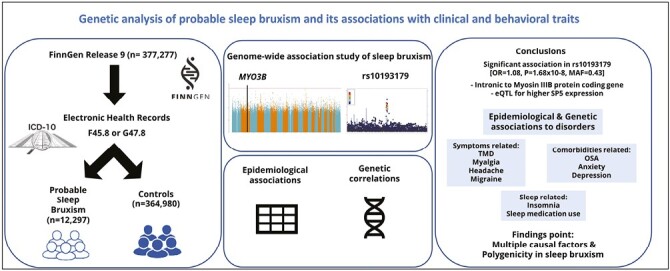

**Statement of Significance**

Sleep bruxism (SB) affects a considerable part of the population, but its clinical and epidemiological correlates have not been previously examined using combined registry data with clinical and genetic epidemiology. Understanding the mechanisms underlying SB is important as it may advise clinical decision making for physicians and dentists. Furthermore, understanding bruxism may allow improved comprehensive management in the presence of both the condition and its comorbidities. Previous studies on SB have provided important symptomatic insight but are mainly questionnaire-based or limited in sample size. This paper builds upon the previous literature clarifying associations with pain and migraine, sleep apnea, insomnia, and psychiatric comorbidities by combining registry data to genetic information.

## Introduction

Bruxism is defined as a repetitive jaw-muscle activity characterized by clenching or grinding of teeth, or by bracing or thrusting of the mandible. Bruxism can occur during sleep (sleep bruxism, SB) or during wakefulness (awake bruxism, AB) [1]. The prevalence estimate for SB ranges from 6% to 8% and AB has a reported prevalence of 30% in large epidemiological studies mainly assessed by self reports in adult populations [2–4]. Even though SB and AB are considered different entities, a cooccurrence of up to 20% has been reported [5] pointing towards shared etiology. In otherwise healthy individuals, bruxism should be considered a behavior rather than a disorder. However, bruxism can be a risk factor or comorbidity for clinical outcomes, or may be a mechanism to keep the airways open or prevent gastric acid from entering the airways, a possible mechanism why SB is more frequently seen in individuals with sleep apnea or gastroesophageal acid reflux [6–11].

Even though the etiology of AB has remained unclear, SB seems to have several underlying factors: psychological including dissatisfaction, stress sensitivity, and anxiety [12–14]. Furthermore, SB has been suggested to be associated with behavioral traits including alcohol, tobacco, and caffeine use. In addition, pharmacological treatment and especially stimulants and psychoactive medications may lead to SB or worsen preexisting SB [15–17]. Finally, SB is closely related to physiological and clinical traits, including sleep disordered breathing and apnea, or reflux disease [18–20].

Primarily, the increased workload caused by AB and SB may cause pain and temporomandibular disorders as well as excessive tooth wear [18]. However, the major clinical implications of direct effects of bruxism are seen at the level of teeth and have direct consequences on the choice and success of dental treatment. Therefore, it is clinically particularly important for the restorative dentist and prosthodontist [21] to also consider the amount of even short term forces produced by SB. Such forces produced by SB may be substantially larger compared to normal occlusal forces of mastication [22]. The clinical relevance of SB is well established and remains an important factor to be taken into account in treatment planning.

We and others have previously shown that underlying genetic variation may contribute to onset and clinical picture of SB [23–25]. However, the biological pathways and symptomatology that can be explored using genetic etiology, has remained unstudied. In the current work, we wanted to build upon the earlier work and take an unbiased approach through genetic information to elucidate the comorbidities and biological mechanisms that lead to SB by examining the genetic determinants and correlates of probable [1] SB in 377 277 individuals with genetic and electronic health record data.

## Methods

In the International Classification of Diseases (ICD), 10th revision, by the World Health Organization [26], bruxism is listed under F45.8 “Other Somatoform Disorders.” The adoption of F45.8 code as the code used to denote bruxism in Finland has been implicated also in a recent study and shown to be diagnosed often by Finnish dentists [27]. To identify individuals with probable SB we extracted ICD-10 codes F45.8 “Other Somatoform Disorders” and G47.8 “Other Sleep Disorders”—another ICD-10 code adopted more recently for SB diagnosis in Finland—from hospital and primary care registries.

As no definitive criteria for AB diagnosis have ever been established [28] and because the signs of SB on the other hand may be quite reliably detected and reported by dentists [29], we can interpret these cases to represent *probable* SB [1], as the diagnoses have been coded by either dentists or medical doctors and requires clinical findings in addition to self-report.

However, as Finland has not adopted a specific ICD-10 code for SB, F45.8 code has also been used to diagnose rare “Other somatoform disorders” and therefore a small portion of our cases may represent psychogenic pruritus, psychogenic paresthesia, or psychogenic menstruation pain instead of probable SB. For this reason, in addition to our main analysis with the logical operation “F45.8 or G47.8,” we decided to perform a separate sensitivity analysis, where we excluded all individuals with ICD-10 codes relating to pruritus, paresthesia or menstruation pain conditions, with the assumption that it is highly unlikely that a patient receives these stigmatizing psychiatric diagnoses at their first visit and therefore would first receive a diagnosis code denoting a physical disorder instead of a psychological one. Consequently, patients with an F45.8 ICD-10 code but also any of the ICD-10 codes under R20 “Disturbances of skin sensation” or L29 “Pruritus” or N94 “Pain and other conditions associated with female genital organs and menstrual cycle” were excluded from the sensitivity analysis dataset.

Also, ICD-10 code G47.8 “Other Sleep Disorders” has been used to represent SB since April 16, 2021 when an update to Finnish national guidelines [30] emerged but before that date code G47.8 has been used to describe a category of rare sleep disorders e.g. Kleine–Levin syndrome or other organic sleep disorders. Therefore, regarding G47.8 code in our sensitivity analysis we decided to also exclude all individuals with the G47.8 ICD-10 code before April 16, 2021, from our dataset.

These exclusion methods resulted in a subset of individuals with F45.8 or G47.8 codes to represent individuals with probable SB (*N* = 10 681) and 348 276 controls and a second genome-wide association analysis (GWAS) was also performed resulting in the same genome-wide significant finding in *MYO3B* (OR = 1.08, *p* = 4.63 × 10^−8^). (Supplementary Figure 1)

### Study cohort

FinnGen (www.finngen.fi/en) is a large biobank-based cohort aiming to genotype 500 000 Finns. FinnGen integrates the genomic data with longitudinal Finnish medical registry information that records healthcare events over the individual’s entire lifespan using unique national personal identification codes. The registry data combines statistics from several registries including hospital and primary care diagnoses (available from 1968 to 2011, respectively) and information from medication purchases registry [31] (available from 1995). Data in the medication purchases register include the date of the purchase, the International Anatomical Therapeutic Chemical classification code indicating the generic name of the drug and the dose prescribed. Over-the-counter drugs or medications given to institutionalized persons are not included in the register. The data from each registry were available from the beginning of the registry until October 11, 2021, except for medication purchases registry, which was available until December 31, 2020.

The FinnGen’s data release R9 used in this study includes 377 277 participants. To identify individuals with probable SB we extracted ICD-10 codes F45.8 “Other Somatoform Disorders” and G47.8 “Other Sleep Disorders” with the logical operation “F45.8 or G47.8” from hospital and primary care registries resulting in a total of 12 297 (3.26% of the total FinnGen R9) participants with probable SB. Individuals only with either ICD-10 code F45.8 or G47.8 amounted to 11 957 and 289, respectively, while 51 individuals had both ICD-10 codes. All FinnGen’s clinical phenotype definitions used in this study are available in Table 1.

**Table 1.** International Classification of Diseases (ICD) Finnish National Version and Anatomical Therapeutic Chemicals (ATC) Classification Codes for Probable Sleep Bruxism and Phenotypes

NAME	ICD-10	ICD-9	ICD-8	ATC
Bruxism	F45.8|G47.8			
TMD	K07.6			
Sleep apnea	G47.3	3472		
Reflux	K21	5301A		
Upper respiratory diseases	J3	470|471|472|473|474| 475|476|477|478	500|501|502|503|50499| 505|506|507|508	
Anxiety	F41.[2–9]	3000A	3000	
Depression	F32|F33	2961|2968|3004		
Clinical insomnia	F51.0|G47.0			
Pain	G50.0|G50.1|G54.6|H57.1| K07.6|M25.5|M54.[0-6]| M54.[8-9]|M79.[0–3]| M79.[6–7]| R10|R51|R52	3501|3502|3536|7231| 7234|7241|7242|7244| 7245|7290|7291|7293X| 7840|7890	35199|71491|7170| 71899|7283| 7285|72870|7288|7289| 78199|78559|7873	
Myalgia	M79.1	7291		
Dorsalgia	M54.[0–6]|M54.[8–9]	7231|7234|7241|7242| 7244|7245	7170|7283|7285|72870| 7288|7289	
Headache	G44		34601	
Migraine	G43	346	346	
Antidepressants				N06AA|N06AB|N06AF| N06AG|N06AX
Hypnotics/sedatives				N05C

TMD, temporomandibular disorders.

### Genotyping and imputation

FinnGen samples were genotyped with Illumina and Affymetrix chip arrays (Illumina Inc., San Diego, and Thermo Fisher Scientific, Santa Clara, CA, USA). The genotyping of new samples is performed on a FinnGen ThermoFisher Axiom custom array at the ThermoFisher genotyping service facility in San Diego. The array consists of 736 145 probes for 655 973 genetic markers. In addition to the core GWAS markers of around 500 000, the array also contains 116 402 coding variants which are enriched in Finland. The legacy samples have previously been genotyped over the years using various generations of Illumina GWAS arrays. All GWAS data are imputed against a Finnish population-specific imputation panel. Genotype calls were made with GenCall and zCall algorithms for Illumina and AxiomGT1 algorithm for Affymetrix data. Chip genotyping data produced with previous chip platforms and reference genome builds were lifted over to build version 38 (GRCh38/hg38) following the protocol described here: dx.doi.org/10.17504/protocols.io.nqtddwn. In sample-wise quality control, individuals with ambiguous gender, high genotype missingness (>5%), excess heterozygosity (±4 standard deviation), and genetically inferred non-Finnish ancestry were excluded. In variant-wise quality control variants with high missingness (>2%), low Hardy–Weinberg equilibrium *P*-value (<1.0 × 10^−6^) and minor allele count < 3 were excluded. Prior imputation, chip genotyped samples were pre-phased with Eagle 2.3.5 with the default parameters, except the number of conditioning haplotypes was set to 20 000. Genotype imputation was done with the population-specific The Sequencing Initiative Suomi (SISu) v4 reference panel. Variant call set was produced with Genomic analyses toolkit (GATK) HaplotypeCaller algorithm by following GATK best-practices for variant calling. Genotype-, sample-, and variant-wise quality control was applied in an iterative manner by using the Hail framework v0.1 and the resulting high-quality whole genome sequenced data for 3775 individuals were phased with Eagle 2.3.5. Post-imputation quality control involved excluding variants with INFO score < 0.7. After quality controlling our data included 20861210 single nucleotide polymorphisms.

### Statistical analyses

Statistical software used was R version 4.2.1. For the epidemiological analyses we used logistic regression to examine the association between probable SB and its risk factors, comorbidities, and medication purchases. Diagnoses and medications were ascertained at death or last follow-up. All analyses were adjusted for age at death or end of follow-up, sex and first 10 principal components (PCs), except the analysis for sex was adjusted for age at death or end of follow-up and first 10 PCs and analysis for age at death or end of follow-up was adjusted for sex and first 10 PCs.

A total of 377 277 samples from FinnGen R9 with 2272 core disease endpoints were analyzed using REGENIE software [32] for genome-wide association analyses (GWAS). All analyses were adjusted for age at death or end of follow-up, sex, genotyping chip, genetic relationship, and first 10 PCs. For probable SB we formed a disease endpoint by combining F45.8 and G47.8 ICD-10 codes with the logical operation “F45.8 or G47.8” and conducted a GWAS in a similar manner.

To estimate genetic correlations between probable SB and its associated traits, we utilized linkage disequilibrium score regression (LDSC) [33]. In our analyses we used LD scores calculated from the 1000 Genomes project European reference panel [34]. We also calculated SNP-based heritability on the liability scale for each trait [35]. For the calculation we used a set of common, well-imputed variants, and retained the single nucleotide polymorphisms which were included in the HapMap 3 reference panel [36]. We used summary statistics for each trait from FinnGen core endpoints and also utilized data from previously published studies from UK Biobank for daytime sleepiness and insomnia [37, 38]. The statistical significance threshold was 5 × 10^−8^ for GWAS analyses and 0.05 in all other analyses.

The Genotype-Tissue Expression (GTEx, https://gtexportal.org/home/) project [39] aims to provide a resource to study human gene expression and regulation and estimate the effect of genetic variants on gene expression. This project will collect and analyze multiple human tissues from donors who are also densely genotyped, to assess the effect of genetic variation on gene expression in a tissue specific manner. By analyzing global RNA expression within individual tissues and treating the expression levels of genes as quantitative traits, variations in gene expression that are highly correlated with genetic variation can be identified as expression quantitative trait loci. We used the equantitative trait loci calculator as provided by GTEx version 8 to analyze *MYO3B* intronic rs10193179.

## Results

### Epidemiological association with probable SB

Using data from FinnGen (*N* = 377 277) we found 12 297 (3.26%) individuals with SB-related ICD-10 codes (F45.8 or G47.8). We first performed a descriptive analysis of the cohort to understand the associations between probable SB and demographic factors. We observed association with younger age (average 51 years vs. 60 years in individuals without probable SB, Tables 2 and 3) and female sex (OR = 2.79 [2.66–2.91]), and with previously implicated clinical comorbidities (Tables 2 and 3). We observed associations with temporomandibular disorders (TMD, OR = 6.75 [6.43–7.09]), sleep apnea (OR = 1.89 [1.79–2.00]), reflux disease (OR = 2.06 [1.94–2.19]) and upper respiratory diseases (OR = 1.82 [1.75–1.89]), pain diagnoses (OR = 2.02 [1.94–2.09]), myalgia (OR = 4.42 [4.11–4.76]), dorsalgia (OR = 1.76 [1.69–1.84]), headache (OR = 2.39 [2.25–2.54]) and migraine (OR = 1.98 [1.87–2.09]), insomnia (OR = 2.30 [2.06-2.58]), psychiatric traits (anxiety and depression, OR = 1.84 [1.73–1.96] and OR = 1.80 [1.73–1.89], respectively) and with medications to manage psychiatric traits and insomnia (antidepressants and sleep medications, OR = 1.91 [1.84–1.98] and OR = 1.59 [1.51–1.66], respectively).

**Table 2.** Descriptive Demographics and Disease Associations With Probable Sleep Bruxism

	All, *N* = 377 277	Bruxism *N* = 12 297	No bruxism *N* = 364 980	OR [95%]	*p*
Sex					
Male	166 407 (44.1%)	2476 (20.1%)	163 931 (44.9%)	1	
Female	210 870 (55.9%)	9821 (79.9%)	201 049 (55.1%)	2.78 [2.66 to 2.91]	<1 × 10^−300^
Age in years (SD)	60.6 (17.9)	51.0 (15.8)	60.9 (17.8)	0.97 [0.97 to 0.98]	<1 × 10^−300^
TMD	13 282 (3.5%)	2607 (21.2%)	10 675 (2.9%)	6.75 [6.43 to 7.09]	<1 × 10^−300^
Sleep apnea	38 998 (10.3%)	1578 (12.8%)	37 420(10.3%)	1.88 [1.78 to 1.99]	5.53 × 10^−110^
Reflux	26 184 (6.9%)	1370 (11.1%)	24 814 (6.8%)	2.05 [1.93 to 2.17]	3.37 × 10^−125^
Upper respiratory diseases	93 935 (24.9%)	4982 (40.5%)	88 954 (24.4%)	1.81 [1.75 to 1.88]	1.33 × 10^−214^
Anxiety	16 887 (4.5%)	1306 (10.6%)	15 581 (4.3%)	1.84 [1.73 to 1.96]	1.37 × 10^−85^
Depression	43 280 (11.5%)	2744 (22.3%)	40 536 (11.1%)	1.80 [1.72 to 1.88]	1.30 × 10^−147^
Clinical insomnia	4214 (1.1%)	338 (2.7%)	3876 (1.1%)	2.29 [2.05 to 2.57]	7.77 × 10^−46^
Pain	171 922 (45.6%)	7857 (63.9%)	164 065 (45.0%)	2.01 [1.94 to 2.08]	2.22 × 10^−283^
Myalgia	7024 (1.9%)	925 (7.5%)	6099 (1.7%)	4.41 [4.10 to 4.74]	<1 × 10^−300^
Dorsalgia	59 438 (15.8%)	2851 (23.2%)	56 587 (15.5%)	1.75 [1.68 to 1.83]	1.14 × 10^−139^
Headache	15 851 (4.2%)	1236 (10.1%)	14 615 (4.0%)	2.38 [2.23 to 2.53]	3.77 × 10^−164^
Migraine	18 477 (4.9%)	1526 (12.4%)	16 951 (4.6%)	1.97 [1.86 to 2.08]	4.13 × 10^−120^
Antidepressants	106 785 (28.3%)	5417 (44.1%)	101 368 (27.8%)	1.90 [1.83 to 1.98]	8.56 × 10^−256^
Hypnotics/sedatives	65 814 (17.4%)	2425 (19.7%)	63 389 (17.4%)	1.58 [1.51 to 1.66]	6.51 × 10^−80^

Correlations with probable sleep bruxism (International Classification of Diseases (ICD)-10 codes F45.8 or G47.8) with known or suspected risk factors and comorbidities. Odds ratios (ORs) and *P*-values have been adjusted for age at death or end of follow-up, sex and first 10 principal components. ICD- and Anatomical Therapeutic Chemicals (ATC) classification codes are presented in Table 1. OR, odds ratio; SD, standard deviation; TMD, temporomandibular disorders.

**Table 3.** Descriptive Demographics and Disease Associations With Probable Sleep Bruxism of Non-related Individuals

	All, *N* = 233 247	Bruxism *N* = 7665	No bruxism *N* = 225 582	OR [95%]	*p*
Sex					
Male	102 853 (44.1%)	1569 (20.5%)	101 284 (44.9%)	1	
Female	130 394 (55.9%)	6096 (79.5%)	124 298 (55.1%)	2.71 [2.57 to 2.87]	8.83 × 10^−261^
Age in years (SD)	60.2 (17.8)	51.1 (15.8)	60.5(17.8)	0.98 [0.97 to 0.98]	<1 × 10^−300^
TMD	8174 (3.5%)	1597 (20.8%)	6577 (2.9%)	6.68 [6.28 to 7.10]	<1 × 10^−300^
Sleep apnea	24 163 (10.4%)	981 (12.8%)	23 182 (10.3%)	1.85 [1.72 to 1.99]	2.29 × 10^−65^
Reflux	15 932 (6.8%)	859 (11.2%)	15 073 (6.7%)	2.09 [1.94 to 2.26]	4.98 × 10^−84^
Upper respiratory diseases	57 977 (24.9%)	3083 (40.2%)	54 894 (24.3%)	1.81 [1.73 to 1.90]	6.10 × 10^−134^
Anxiety	10 915 (4.7%)	840 (11.0%)	10 075 (4.5%)	1.87 [1.73 to 2.02]	4.20 × 10^−58^
Depression	27 279 (11.7%)	1734 (22.6%)	25 545 (11.3%)	1.81 [1.71 to 1.91]	2.08 × 10^−95^
Clinical insomnia	2685 (1.2%)	219 (2.9%)	2466 (1.1%)	2.34 [2.03 to 2.70]	1.13 × 10^−31^
Pain	105 729 (45.3%)	4893 (63.8%)	100 836 (44.7%)	2.02 [1.92 to 2.12]	1.04 × 10^−180^
Myalgia	4354 (1.9%)	586 (7.6%)	3768 (1.7%)	4.47 [4.08 to 4.90]	3.46 × 10^−222^
Dorsalgia	36 096 (15.5%)	1738 (22.7%)	34 358 (15.2%)	1.73 [1.64 to 1.83]	5.14 × 10^−83^
Headache	9601 (4.1%)	800 (10.4%)	8801 (3.9%)	2.56 [2.37 to 2.77]	9.60 × 10^−125^
Migraine	11 237 (4.8%)	910 (11.9%)	10 327 (4.6%)	1.92 [1.79 to 2.07]	3.77 × 10^−68^
Antidepressants	66 701 (28.6%)	3435 (44.8%)	63 266 (28.0%)	1.94 [1.85 to 2.03]	2.01 × 10^−169^
Hypnotics/sedatives	40 627 (17.4%)	1558 (20.3%)	39 069 (17.3%)	1.62 [1.52 to 1.72]	2.79 × 10^−56^

Correlations with probable sleep bruxism (International Classification of Diseases (ICD)-10 codes F45.8 or G47.8) with known or suspected risk factors and comorbidities of a subset where only unrelated Finnish ancestry individuals of Finngen R9 were included. Odds ratios (ORs) and *P*-values have been adjusted for age at death or end of follow-up, sex, and 10 principal components. ICD- and Anatomical Therapeutic Chemicals (ATC) classification codes are presented in Table 1. OR, odds ratio; SD, standard deviation; TMD, temporomandibular disorders.

### Genetic association with probable SB

**Figure 1.**
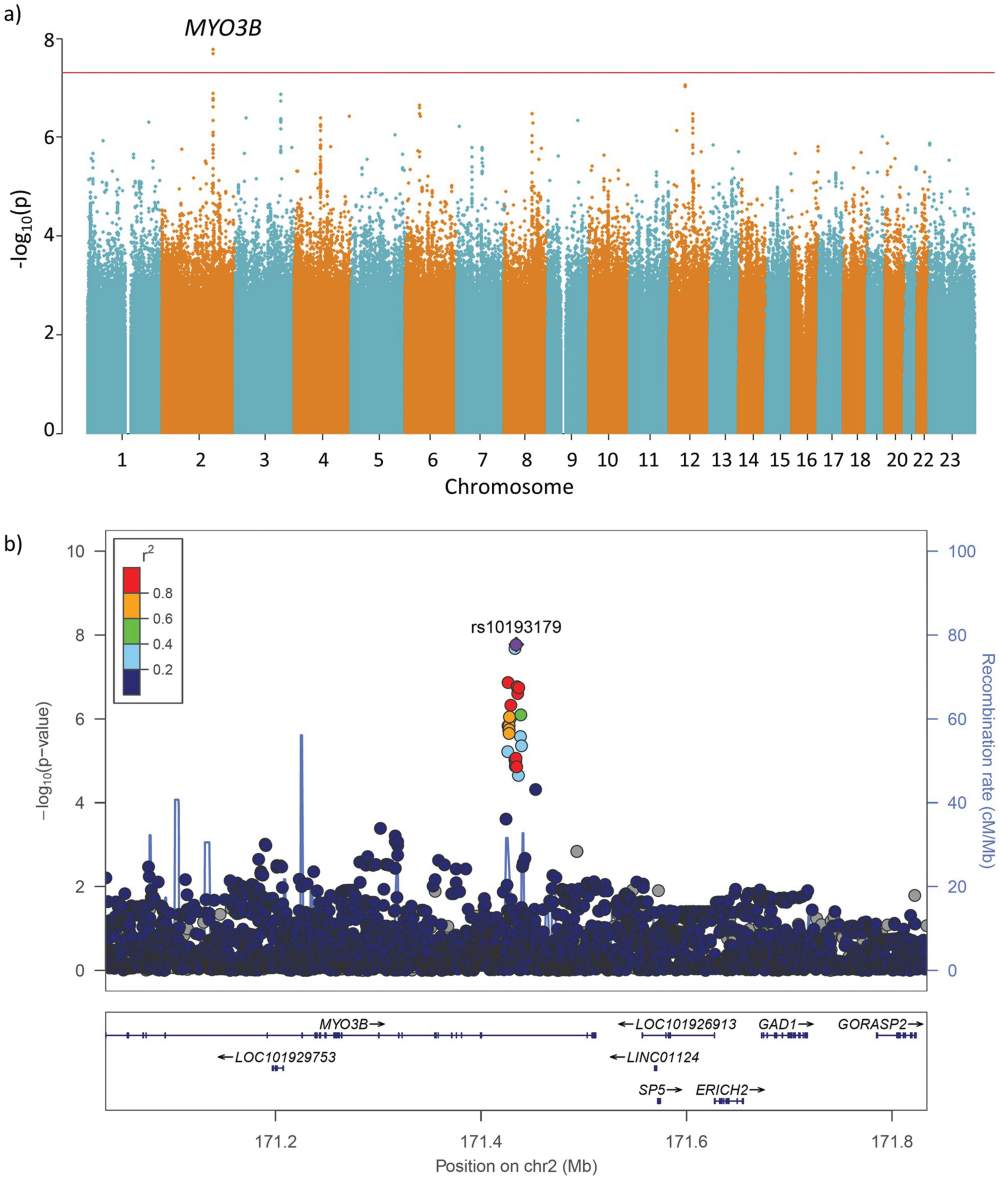
Genome-wide analysis of probable sleep bruxism**.** (a) Manhattan plot for probable sleep bruxism; 12 297 cases and 364 980 controls. *X*-axis represents chromosomal position for each variant. *Y*-axis shows the − log_10_(P) value. The horizontal line indicates the genome-wide significance threshold of P = 5 × 10^−8^. *MYO3B* = Myosin 3B (b) Locus Zoom plot of the main finding rs10193179 (purple diamond) in chromosome 2. The *x*-axis represents chromosome position (hG38) with annotated genes found within the region and the *y*-axis shows − log_10_(*p*) value.

To explore the potential biological mechanisms and understand underlying pathology we performed a GWAS for probable SB in the same 377 277 individuals, where 12 297 participants had probable SB diagnosis and 364 980 were controls (Figure 1). The genome-wide scan analysis produced one significant association at rs10193179 intronic to *Myosin IIIB* (*MYO3B)* gene (OR = 1.08 [1.05–1.11], P = 1.68 × 10^−8^, minor allele frequency = 0.43).

As genetic association results do not necessarily implicate a causal gene, we explored the biological correlates at the *MYO3B* region. We observed that the intronic rs10193179 A-allele was an expression quantitative trait locus (eQTL) for higher *Sp5 Transcription Factor* (*SP5*) expression across tissues including artery and pituitary (*p* = 1.6 × 10^−7^ and *p* = 1.6 × 10^−3^, respectively). Earlier *SP5* mouse knockout has described only two affected features and these include increased grip strength and hyperactivity [40, 41].

To estimate the robustness of our phenotype, we performed a sensitivity analysis described in the methods section. These findings are described in Supplementary Material and show a significant association at the same locus of *MYO3B* (rs10193179, *p* = 4.65 × 10^−8^). (Supplementary Figure 1)

### Genetic correlation analysis with comorbid phenotypes and sleep phenotypes

In order to test the genetic correlation between probable SB and the risk factors implicated from earlier studies and also our own epidemiological analysis, we performed a genetic correlation analysis. We discovered that similar to earlier work, the genetic analysis supported the association between probable SB and depression, as seen by both depression medication and by diagnoses of depression (Table 4). Furthermore, we observed a significant association with self-reported insomnia and sleep medication use, whereas clinically diagnosed insomnia did not remain statistically significant after correction for multiple testing. In addition, we observed a significant association of probable SB with sleep apnea and reflux disease. It is noteworthy that pain diagnoses are associated with probable SB. Consequently, general pain diagnoses and specific pain diagnoses captured by headache are both associated with probable SB. Finally, a significant contribution from TMD—disorders of the jaw muscles, temporomandibular joints, and the nerves associated with chronic facial pain—associated with probable SB. It is important to note that the diagnosis code for TMD does not include SB-related codes. However, the association is expected as the mechanical strain produced by SB events may reach pathological levels and contribute to disorders in related areas resulting in TMD. The liability-scale heritability estimate of probable SB was 5.4% with a standard error of 1.2%.

**Table 4.** Genetic Correlation Between Probable Sleep Bruxism and Its Risk Factors and Comorbidities

Trait	Genetic correlation (rg)	Standard error (se)	Z	*p*	*p* adj.	*h*_*l*_^2^ (se)
TMD	0.725	0.126	5.779	7.53 × 10^−09^	1.20 × 10^−07^	8.3% (1.3%)
Sleep apnea	0.319	0.072	4.426	9.59 × 10^−06^	1.53 × 10^-−04^	15.9% (0.9%)
Reflux	0.633	0.111	5.686	1.30 × 10^−08^	2.08 × 10^−07^	9.7% (0.7%)
Upper respiratory diseases	0.490	0.081	6.077	1.22 × 10^−09^	1.95 × 10^−08^	7.9% (0.6%)
Anxiety	0.428	0.109	3.935	8.34 × 10^−05^	1.33 × 10^−03^	14.0% (1.2%)
Depression	0.501	0.091	5.529	3.22 × 10^−08^	5.15 × 10^−07^	10.0% (0.7%)
Clinical insomnia	0.311	0.144	2.161	0.031	0.496	17.6% (3.7%)
Insomnia*	0.205	0.064	3.185	1.45 × 10^−03^	0.023	16.6% (0.6%)
Sleepiness*	0.138	0.064	2.171	0.030	0.480	4.8% (0.2%)
Pain	0.505	0.073	6.903	5.11 × 10^−12^	8.18 × 10^−11^	7.6% (0.4%)
Myalgia	0.758	0.158	4.808	1.52 × 10^−06^	2.43 × 10^−05^	8.8% (0.2%)
Dorsalgia	0.381	0.072	5.324	1.02 × 10^−07^	1.63 × 10^−06^	10.8% (0.6%)
Headache	0.517	0.101	5.124	2.99 × 10^−07^	4.78 × 10^−06^	15.4% (1.7%)
Migraine	0.442	0.094	4.704	2.55 × 10^−06^	4.08 × 10^−05^	13.2% (1.1%)
Antidepressants	0.465	0.076	6.148	7.85 × 10^−10^	1.26 × 10^−08^	6.9% (0.4%)
Hypnotic/sedatives	0.462	0.079	5.861	4.60 × 10^−09^	7.36 × 10^−08^	8.1% (0.4%)

Genetic correlations with probable sleep bruxism and known or suspected risk factors and comorbidities. * indicates that summary statistics are from previous studies utilizing UK Biobank data [37, 38]. *h*_*l*_^2^, liablity-scale heritability; TMD, temporomandibular disorders; *p* adj., bonferroni adjusted *P*-value.

## Discussion

In this study we describe a genetic association analysis for probable SB and identify *MYO3B* as a potential risk locus. Furthermore, our findings implicate reflux disease, sleep apnea, insomnia, medications and especially TMD, and pain diagnoses as key comorbidities with probable SB with both phenotypic associations and genetic correlations. Furthermore, our genetic correlation findings and epidemiological associations in the study cohort are in line with earlier results from previous studies and support the multifactorial nature of SB.

In this study we discovered a genetic association at the *MYO3B* locus. *MYO3B* gene encodes for Myosin-IIIb protein. The best described function of the protein is its involvement in hearing [42] and in vision [43, 44] and participates in responding to mechanosensory input in these cells [45]. Such a mechanism may play a role also in probable SB, as Myosin-IIIb protein has been implicated in actin filament function and encodes one of the class III myosins [46]. Myosin-IIIb protein similar to other myosins is an ATPase and gets activated by actin. Myosins are notably important in muscles [47] and the possible mechanisms might include muscle contraction during bruxism event. In addition, this class of myosins are characterized by an amino-terminal kinase domain and shown to be present in photoreceptors [43, 44], which are notably important overall in sleep as photoreceptors convey light signal to the suprachiasmatic nucleus [48]. However, careful follow-up work of the genetic signal, the possible target genes and downstream biological mechanisms are needed to fully understand and characterize the biological mechanisms between the locus association and bruxism. However, it is also possible that the closest gene is not the causal gene for genetic association so that the variant association at *MYO3B* locus contains a regulatory element that is relevant for another gene and represents an association related to some other mechanism in this area of the genome.

Our follow-up analysis using gene expression data showed that the risk variant rs10193179 associated with gene expression levels of SP5 gene. These findings raise a possibility of *SP5* as an additional likely contributing gene to disease pathology at the *MYO3B* region. Aligning with the clinical picture of SB, knockout mice deficient for *SP5* manifest with increased grip strength and hyperactivity [40, 41]. Therefore, the genetic findings from the *MYO3B* locus implicate possibly two genes as likely targets for understanding the mechanisms behind probable SB, although the exact mechanism needs to be validated in larger genetic studies in humans and followed up carefully in cellular models.

One of the strongest findings in the present study was the genetic and epidemiological correlates of probable SB with previously implicated risk factors. In particular, reflux disease and sleep apnea were associated with probable SB. This is interesting as currently the paradigm is shifting towards considering SB also as a protecting reflex to keep the airways open during apnea events and perhaps even to protect from acidic stomach contents in reflux disease [7, 9, 21, 49]. Furthermore, our sleep associations with insomnia and daytime sleepiness may reflect poor sleep quality from SB itself [50], from psychological factors like anxiety or depression, or from the closely related comorbidities such as sleep apnea which may increase SB behavior. Causality between the associations; however, remains elusive and may be addressed in future iterations of this work.

Females were overrepresented in our probable SB cases which may relate to women seeking medical attention due to and reporting the consequences—most likely pain related—of SB more readily whilst men possibly remain undiagnosed. In addition, the FinnGen cohort itself includes more women than men (55.9%). It is noteworthy that pain diagnoses were strongly associated with probable SB and included both general pain diagnoses, and specific pain diagnoses captured by headache. This finding had also relatively strong effect sizes, particularly for myalgia (OR = 4.42) and headache (OR = 2.39) in the epidemiological analysis, and over 0.50 genetic correlation with headache and pain clearly implicated shared disease etiology. Overall, these findings highlight the impacts that SB can cause at individual level and suggest that probable SB should be treated and managed when possible. We anticipate that the majority of bruxism cases reflect SB but a subset of patients may have concomitant AB and we cannot clinically separate them with the codes available from ICD, so it is possible that for example comorbidities such as myofascial pain may also be related to AB. Therefore, it will be important to characterize the overlap and differences between AB and SB in future studies.

Finally, diseases involving the jaw muscles and joints (temporomandibular disorders) are associated with probable SB. While currently correlative, the findings implicate that many individuals with primarily temporomandibular complaints have SB. This finding may have clinical implications for patient treatment and needs to be examined further.

### Limitations

Our analysis estimates the lifetime risk between probable SB and comorbidities using logistic regression without incorporating temporal information. However, the question of whether comorbidities and diagnoses or medication prescriptions precede or follow the probable SB diagnosis would be informative. To estimate the temporal relationships between comorbidities and probable bruxism, we performed a Cox proportional hazards analysis. However, the data did not fulfill the assumptions required for testing Cox models so that the global *P*-value was significant even when using age as the timescale and when adjusting for demographic factors and population structure. Therefore, we did not incorporate temporal Cox proportional hazards analyses in this study.

Our findings should be interpreted in the light of the same limitations as any electronic health record-based study; the diagnosis of SB is code based. Therefore, with the current diagnosis system in Finland we were unable to differentiate between AB and SB. The role of possible concomitant AB in aggravating e.g. musculoskeletal symptoms cannot be excluded as the prevalence of AB has been reported to be relatively high in self-report based studies [2], although a paucity of data regarding AB has been notified [21]. In addition, we did not separate codes by medical specialities and did not perform sensitivity analyses with codes administered solely by dentists or other specialities. Furthermore, FinnGen is enriched for clinical diagnoses and while powerful for genetic studies the cohort does not reflect the population structure of Finland, and consequently the values reported in the epidemiological analyses should not be interpreted as population averages.

The Finnish ICD-10 classification does not have a specific code used solely for SB and therefore the codes F45.8 and G47.8 may include a small fraction of diagnoses that are not related to, and thus do not reflect, SB. In addition, due to national characteristics of diagnosis of AB and SB, the diagnosis codes of F45.8 and G47.8 are not directly portable to other countries and any replication efforts should be done with either questionnaire data or with those ICD codes that will be available for the respective cohorts.

With the secondary use of EHR data, there is a need for validation of phenotypes, such as bruxism, to ensure that the codes match diagnoses. Furthermore, while earlier studies show validation analysis between SB in population based samples [27], in our analysis we did not perform separate validation with chart data but relied on the earlier observations and clinical recommendations. This approach may induce heterogeneity in our analysis that we cannot directly measure in the scope of our own analysis.

## Conclusion

Our study provides a large-scale genetic framework to understand risk factors for probable SB and suggests potential biological mechanisms. Furthermore, our work strengthens the important earlier work that highlights SB as a trait that affects multiple axes of health. As a part of this study, we provide genome-wide summary statistics that we hope will be useful for the scientific community researching SB.

## Supplementary Material

zsad107_suppl_Supplementary_Data

zsad107_suppl_Supplementary_Materials

## Data Availability

As a part of this study, we provide genome-wide summary statistics. The Finnish biobank data can be accessed through the Fingenious® services (https://site.fingenious.fi/en/) managed by FINBB.
