# Supplementary material for: Genetic analysis of probable sleep bruxism and its associations with clinical and behavioral traits
Source: Sleep. 2023 Apr 26;46(10):zsad107. doi: 10.1093/sleep/zsad107 (PMC10566239; doi:10.1093/sleep/zsad107)
Supplement: zsad107_suppl_Supplementary_Materials [file zsad107_suppl_supplementary_materials.docx]

**Genetic analysis of probable sleep bruxism and its associations with clinical and behavioral traits**

Tommi Strausz^1^, Satu Strausz^1,2^, FinnGen, Tuula Palotie^3,4^, Jari Ahlberg^3^, Hanna M. Ollila^1,5,6,7^

1. Institute for Molecular Medicine Finland, Helsinki Institute of Life Science, University of Helsinki, Helsinki, Finland
2. Department of Genetics, Stanford University School of Medicine, Stanford, California, USA
3. Department of Oral and Maxillofacial Diseases, Head and Neck Center, Helsinki University Hospital, Helsinki, Finland
4. Orthodontics, Department of Oral and Maxillofacial Diseases, Clinicum, Faculty of Medicine, University of Helsinki, Helsinki, Finland
5. Broad Institute of MIT and Harvard, Cambridge, Massachusetts, USA
6. Center for Genomic Medicine, Massachusetts General Hospital, Boston, USA
7. Anesthesia, Critical Care, and Pain Medicine, Massachusetts General Hospital and Harvard Medical School, Boston, USA

Corresponding author: Hanna M. Ollila

PL 20 (Tukholmankatu 8), 00014, Finland

[hanna.m.ollila@helsinki.fi](mailto:hanna.m.ollila@helsinki.fi)

Supplementary materials


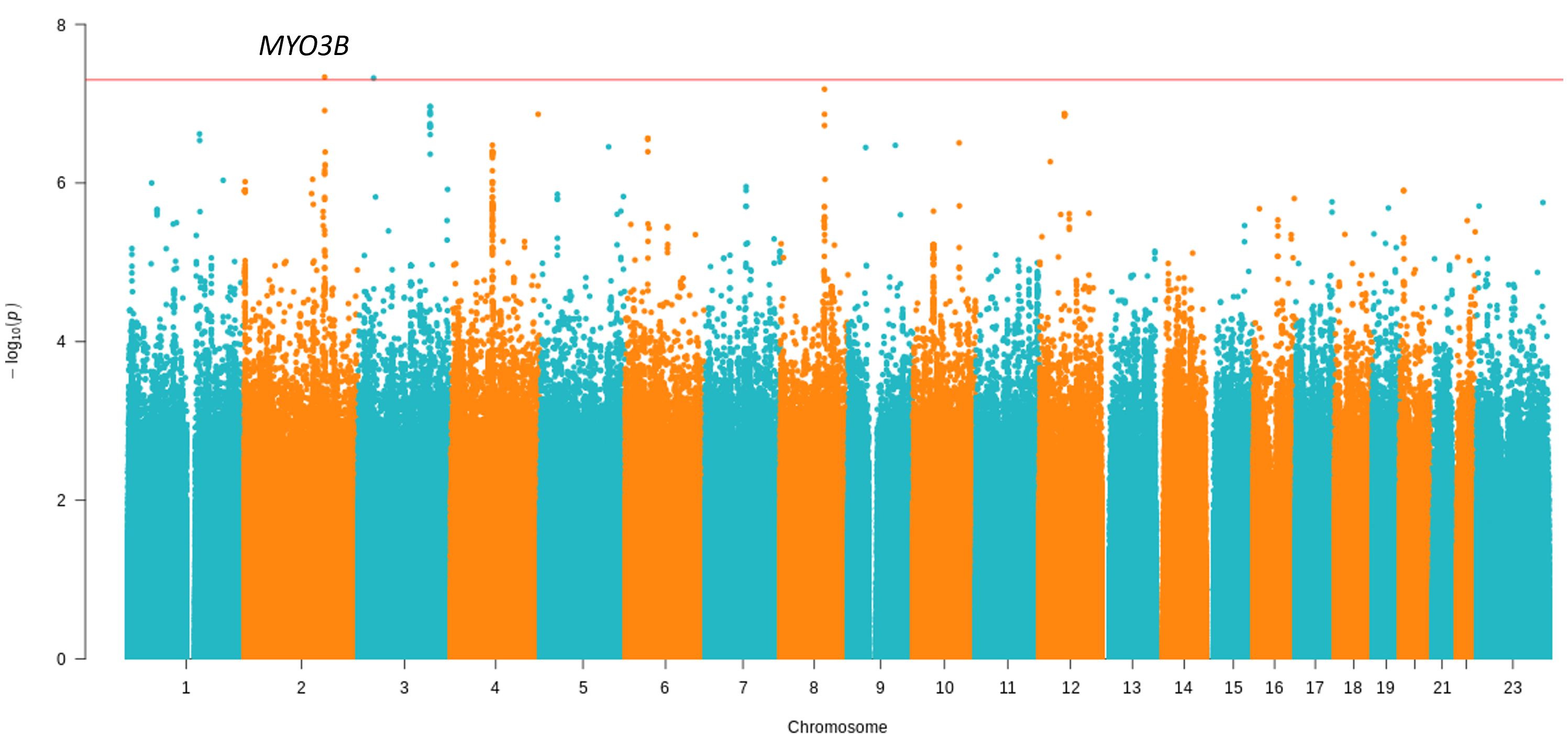
 ***Supplementary Figure 1.*** *Manhattan plot for the sensitivity analysis of probable sleep bruxism; 10,681 cases and 348,276 controls. X-axis represents chromosomal position for each variant. Y-axis shows the −log_10_(P) value. The horizontal line indicates the genome-wide significance threshold of P = 5 × 10^−8^. MYO3B=Myosin 3B*


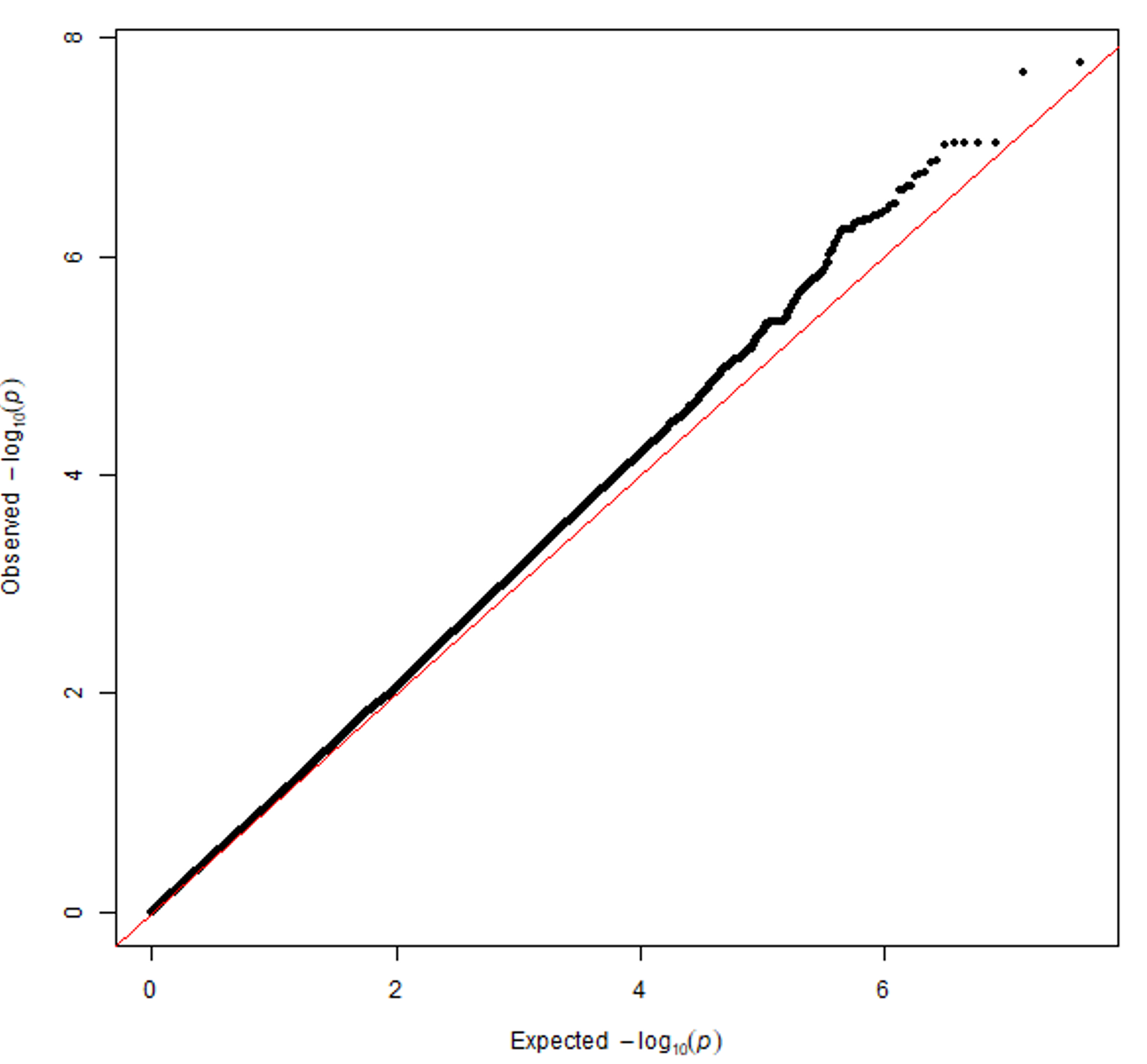


**Supplementary Figure 2.** Quantile-quantile plot for the main genome-wide analysis of probable sleep bruxism with 12,297 cases and 364,980 controls. Lambda 1.04. X-axis shows the expecteded −log_10_(P) value. Y-axis shows the observed −log_10_(P) value.


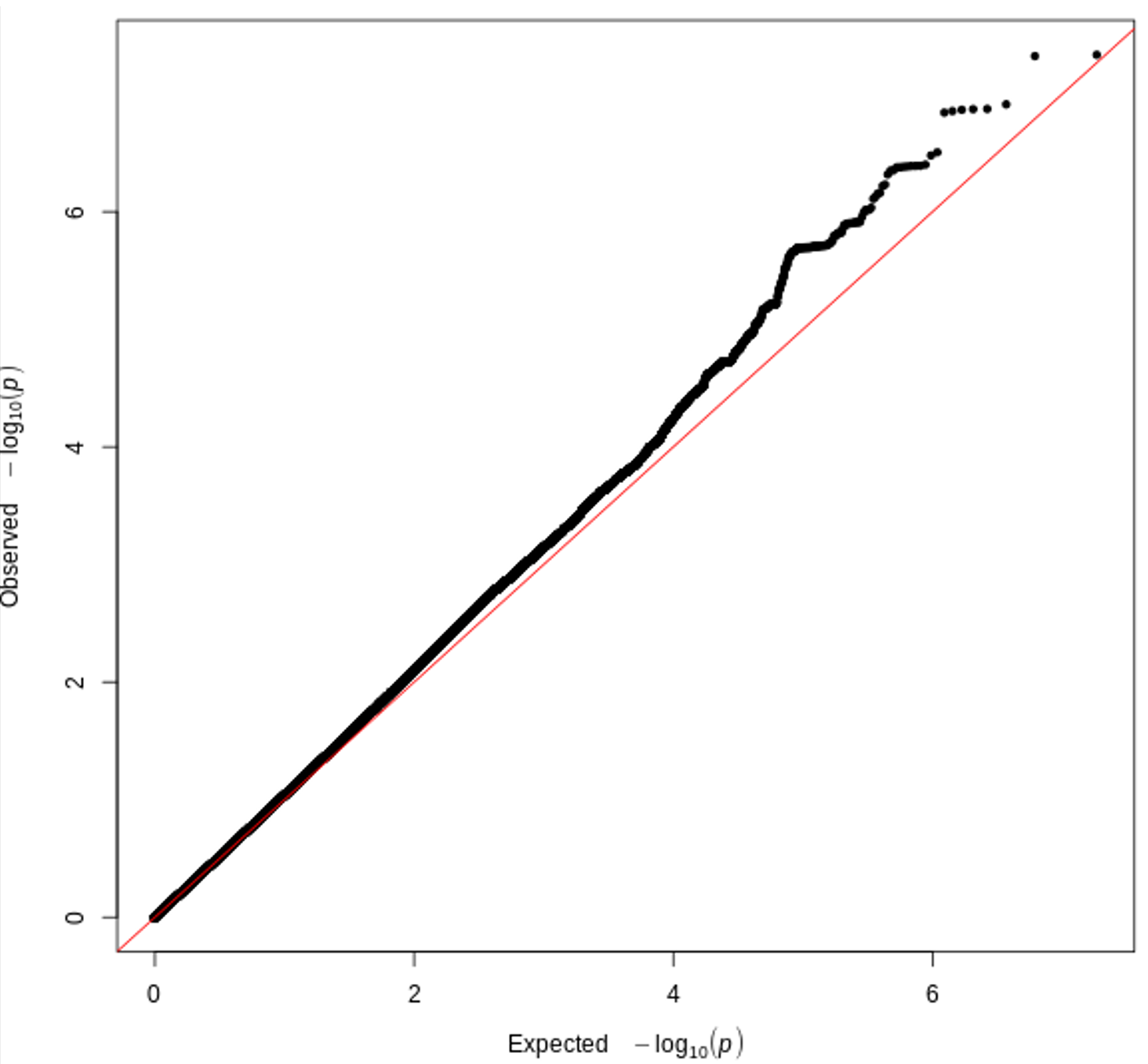


**Supplementary Figure 3.** Quantile-quantile plot for sensitivity analysis of probable sleep bruxism; 10,681 cases and 348,276 controls. Lambda 1.04. X-axis shows the expecteded −log_10_(P) value. Y-axis shows the observed −log_10_(P) value.
